# Update and harmonisation of guidance for the management of diabetic ketoacidosis in children and young people in the UK

**DOI:** 10.1136/bmjpo-2021-001079

**Published:** 2021-06-04

**Authors:** Charlotte EM Rugg-Gunn, Mark Deakin, Daniel B Hawcutt

**Affiliations:** 1 University of Liverpool School of Medicine, Liverpool, UK; 2 Department of Women's and Children's Health, University of Liverpool, Liverpool, UK; 3 Alder Hey Children’s Hospital, Liverpool, UK; 4 NIHR Alder Hey Clinical Research Facility, Liverpool, UK

**Keywords:** resuscitation, diabetes

## Abstract

Diabetic ketoacidosis (DKA) is a life-threatening complication of type 1 diabetes mellitus. Careful and timely intervention is required to optimise glycaemic control and reduce the risk of mortality and devastating complications. Of these, cerebral oedema is the leading cause of death, with a mortality rate of approximately 25%. This article highlights the recent updates to UK fluid therapy guidelines for DKA and provides clinical context for the benefit of paediatricians and junior doctors in light of this new guidance.

**Key messages**

- Updates to two UK guidelines in 2020 has improved concordance, with 0.9% sodium chloride and the Holliday-Segar formula used as a gold standard to calculate fluid maintenance requirement.
- Minor differences remain, such as the maximum weight for maintenance and the stratification of diabetic ketoacidosis or dehydration severity.
- Careful monitoring and adherence to these national guidelines is recommended and will hopefully contribute to a reduction in deaths secondary to cerebral oedema (CO).
- Further research is required to evaluate the efficacy of these new guidelines, and better understand the pathophysiology and risk factors for developing CO.

## Introduction

Diabetic ketoacidosis (DKA) is a potentially life-threatening metabolic complication of type 1 diabetes mellitus. Despite interventions to reduce the incidence of DKA, the National Paediatric Diabetes Audit for 2018/2019 showed that overall 20.9% of all newly diagnosed patients in the United Kingdom (UK) presented in DKA, although there is considerable regional variability.^1^

**Figure 1:**
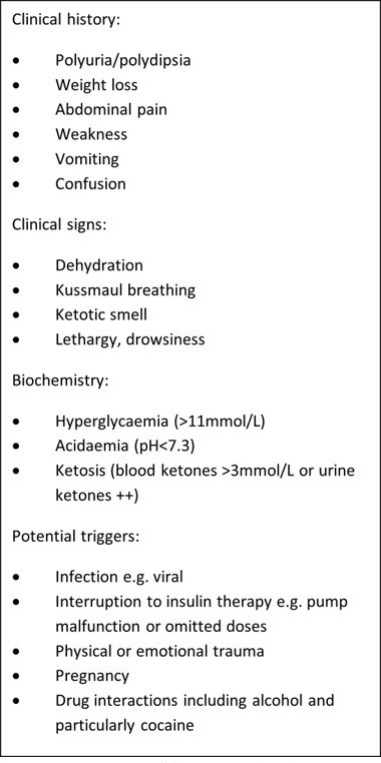
Key signs and symptoms of diabetic ketoacidosis, adapted from Schnabel and Hedrich.^1^

DKA is characterised by uncontrolled hyperglycaemia, ketosis and subsequent metabolic acidosis. As highlighted in figure 1, the clinical picture can develop insidiously due to a variable constellation of non-specific systemic signs and symptoms, including polyuria, polydipsia, weight loss, fatigue, vomiting and abdominal pain, ultimately leading to confusion, coma and death, if untreated.^2 3^ Due to the ambiguity of the presenting clinical features in the early stages, delays in diagnosis are common.^4^

Globally, the International Society for Paediatric and Adolescent Diabetes (ISPAD) guidelines are widely used for management of paediatric DKA.^5^ However, in the UK, it is guided by the integrated care pathways of either the British Society for Paediatric Endocrinology and Diabetes (BSPED) or the National Institute for Health and Care Excellence (NICE). The BSPED guideline evolved from the NICE guideline in 2009 due to concerns over limited evidence that was used for its development. While there were a number of dual-serving board members, the input and additional evidence by the BSPED special interest group in DKA was thought to increase its safety.^6^ Both guidelines cover the diagnosis and management of type one and two diabetes in children and young people (CYP) aged under 18 years.^7^ Areas of particular benefit within a secondary care setting are centred on the requirements for fluid therapy, given the importance of resuscitation in preventing complications. Due to the difference in perceived risk of cerebral oedema (CO) due to rapid fluid administration, there was discordance between the NICE and BSPED 2015 guidelines.

Specifically, the NICE 2015 guidelines were more conservative with fluid administration, in order to avoid rapid changes in osmolality. The BSPED guidelines permitted a more generous fluid allowance, and this raised concerns about precipitating CO. The conflicting advice generated challenges in the emergency setting, with arbitrary site-specific preference rather than evidence-based medicine taking precedence. In addition to generating uncertainty of treatment pathways increasing the risk of clinical error, the regional variability hampered service evaluation and audit of clinical practice.

Unfortunately, there is little data to establish differences in their efficacy, especially surrounding the risk of CO in the paediatric population. However, inadequate resuscitation is likely to increase the risk of brain injury and thus must be avoided. Further research on a national scale is required to assess this.

This article aims to highlight the considerable alterations made to the two main UK guidelines. Alongside these updates, this article will provide clinical context to paediatricians and junior doctors who are likely to treat these vulnerable groups.

## Updated guidance

In January 2020, the BSPED published new guidance for the management of children with DKA.^6^ This integrated care pathway migrated further from the model of restrictive fluid replacement towards an even more flexible approach for resuscitative and maintenance fluids. The rationale for this was based on new evidence from the Pediatric Emergency Care Applied Research Network (PECARN) DKA Fluid trial, which suggested that there was no significant difference in outcomes between rapid and slower fluid administration.^8^ Additionally, it showed that the initial conscious level was closely related to pH and weakly to age, but not to blood glucose or plasma sodium level.^8 9^ While this appears to demonstrate that cerebral function is related to the severity of acidosis even in the absence of drivers of CO, it does not negate previous evidence that fluid shift may also contribute towards CO.^10 11^

Furthermore, there are clear risks associated with inadequate fluid replacement. A hypovolaemic state will result in systemic hypotension and ultimately, cerebral hypoperfusion which, particularly in the context of acidosis, will increase the risk of a brain injury.^6^

In 2008, following three deaths from CO, the South Thames Retrieval Service introduced a ‘restrictive fluid’ DKA guideline in the region. Subsequently, no deaths have been reported, but this is insufficient evidence on which to make definitive recommendations or guidelines.^12^

More recently, in December 2020, NICE released updated guidance on the management of DKA in CYP, which was broadly concordant with BSPED, particularly with respect to more liberal fluid restriction. This was prompted by new evidence identified by NICE’s surveillance team in a review of 12 studies set to determine optimal fluid therapy in CYP with DKA. The fluid protocols followed in the PECARN study were fundamental to these updates,^8^ as ‘neither the rate of administration nor the sodium chloride content of intravenous fluids significantly influenced neurologic outcomes in children with DKA’.^8^ Therefore, restrictions to the fluid administration, which were recommended in the 2015 NICE guidance, ‘were not necessarily required’.^13^ Additionally, as with the BSPED and ISPAD guidelines, the updated NICE guidance was amended to include the Holliday-Segar formula, used to calculate fluid maintenance.^6 7^ Updates to both the BSPED and NICE guidelines in 2020 have a more permissive fluid rate than in the previous DKA fluid therapy guidelines.

Both BSPED and NICE recommend the following fluid maintenance requirements, using 0.9% sodium chloride without added glucose, and the Holliday-Segar formula:

- 100 mL/kg for the first 10 kg (0–10 kg body weight).
- 50 mL/kg for the second 10 kg (10–20 kg body weight).
- 20 mL/kg for each subsequent kilogramme (>20 kg body weight).
- When calculating the total fluid replacement, subtract any initial bolus volumes from the total fluid deficit (unless the child or young person is in shock).

For insulin and electrolyte replacement requirements, both recommend^6 7^:

- The addition of 40 mmol/L potassium chloride to all fluids (apart from the initial intravenous bolus) unless the patient is anuric or hyperkalaemic. Include this before starting the insulin infusion if hypokalaemia is observed at presentation.
- 0.9% sodium chloride should be used without added glucose unless the plasma glucose is <14 mmol/L.
- Initially assess for the presence of hyponatraemia, continue to monitor throughout treatment and treat as soon as blood glucose falls. Monitor sodium levels throughout treatment and calculate sodium initially to identify if hyponatraemia is present. This should be treated as soon as blood glucose falls. It is important to monitor as hyponatraemia and rapidly increasing sodium levels can both be signs of CO.
- Do not give intravenous sodium bicarbonate to CYP with DKA unless they have compromised cardiac contractility caused by life-threatening hyperkalaemia or severe acidosis, and you have discussed with paediatric intensivist.
- A soluble insulin infusion of between 0.05 units/kg/hour and 0.1 units/kg/hour, 1–2 hours after intravenous fluids have been commenced. BSPED stated that the 0.05 units/kg/hour should be sufficient unless the patient is suffering from severe DKA or is an adolescent. If they are attached to a ‘continuous subcutaneous insulin infusion’, disconnect when starting intravenous insulin therapy. For patients already on long-acting insulin, it may be continued, and in new patients consider commencing long-acting subcutaneous insulin alongside intravenous. Similarly, the NICE guidelines state that continuing subcutaneous basal insulin in a child or young person who was using a basal insulin before DKA may be continued in discussion with a diabetes specialist.

Although these guidelines now have greater cohesion, a number of minor differences remain^1 6^:

- Regarding fluid replacement, NICE have included two levels of severity of DKA, while BSPED have subdivided the stratification to include three discrete categories for severity and subsequent dehydration status, as outlined in table 1.
  - A difference remains between the maximum weight considered by the guidelines when calculating fluid deficit. Given that the maximum weight considered for each is 75 kg for NICE and 80 kg for BSPED, the maximum difference in deficit fluids could be 1850 mL (5%×75 kg=3750 mL for NICE compared with 7%×80 kg=5600 mL for BSPED).
- For patients who are clinically dehydrated, but not in shock, an intravenous 10 mL/kg bolus of 0.9% sodium chloride is recommended on admission. This should be administered over 30 min (NICE) or 60 min (BSPED). This volume is included in the fluid replacement when calculating the total fluid deficit. NICE recommend discussing with a paediatrician experienced in DKA management before giving more than one intravenous bolus. A second bolus should only be considered if needed to improve tissue perfusion and this must be done after reassessing their clinical status.
  - A delicate balance between increasing the risk of osmolar shift secondary to rapid fluid administration, with the risk of dehydration and subsequent CO due to slower fluid administration, is the foundation for the initial discrepancy between the two guidelines. Guidance from the 2018 ISPAD consensus states that this should be administered between 30 min and 60 min, which falls between the two UK guidelines.^5^ Therefore, aiming to administer the fluid rapidly but observing for signs of potential deterioration and adjusting the rate accordingly will provide the best outcome for patients.
- In the presence of hypovolaemic shock, both NICE and BSPED recommend an intravenous 0.9% sodium chloride bolus of 20 mL/kg. BSPED recommend repeating these infusions over 15 min to a maximum of 40 mL/kg, before considering the introduction of inotropes, while NICE recommends administering it as soon as possible. In both guidelines, the bolus for shock is not subtracted from total fluid deficit.
  - While the nomenclature varies between these two guidelines, in practice, it is likely that these will represent similar time frames. Administering a 20 mL/kg 0.9% sodium chloride bolus to treat shock as quickly as possible should provide the best therapy for the patient.
- When calculating fluid replacement, the NICE guidelines recommend a maximum weight of 75 kg, whereas BSPED recommend a maximum weight of 80 kg or 97th centile weight for age (whichever is lower).
  - This discrepancy is unlikely to be encountered on a frequent basis in general paediatric practice. However, if the weight greatly exceeds 75 kg, it may be more appropriate for young people to be managed according to adult guidelines due to their increased body habitus. Additionally, identifying a CYP on the 97th centile can be difficult, especially in an emergency setting, and, therefore, this may be missed.

**Table 1:** Comparison of NICE and BSPED DKA classification and dehydration status

Guidelines	Severity of DKA	Dehydration
NICE	Mild–moderate: blood pH≥7.1 Severe: blood pH<7.1	5% 10%
BSPED	Mild (venous pH: 7.2–7.29 or bicarbonate: <15 mmol/L) Moderate (venous pH: 7.1–7.19 or bicarbonate: <10 mmol/L) Severe (venous pH: <7.1 or bicarbonate: <5 mmol/L)	5% 7% 10%

BSPED, British Society for Paediatric Endocrinology and Diabetes; DKA, diabetic ketoacidosis; NICE, National Institute for Health and Care Excellence.

**Figure 2:**
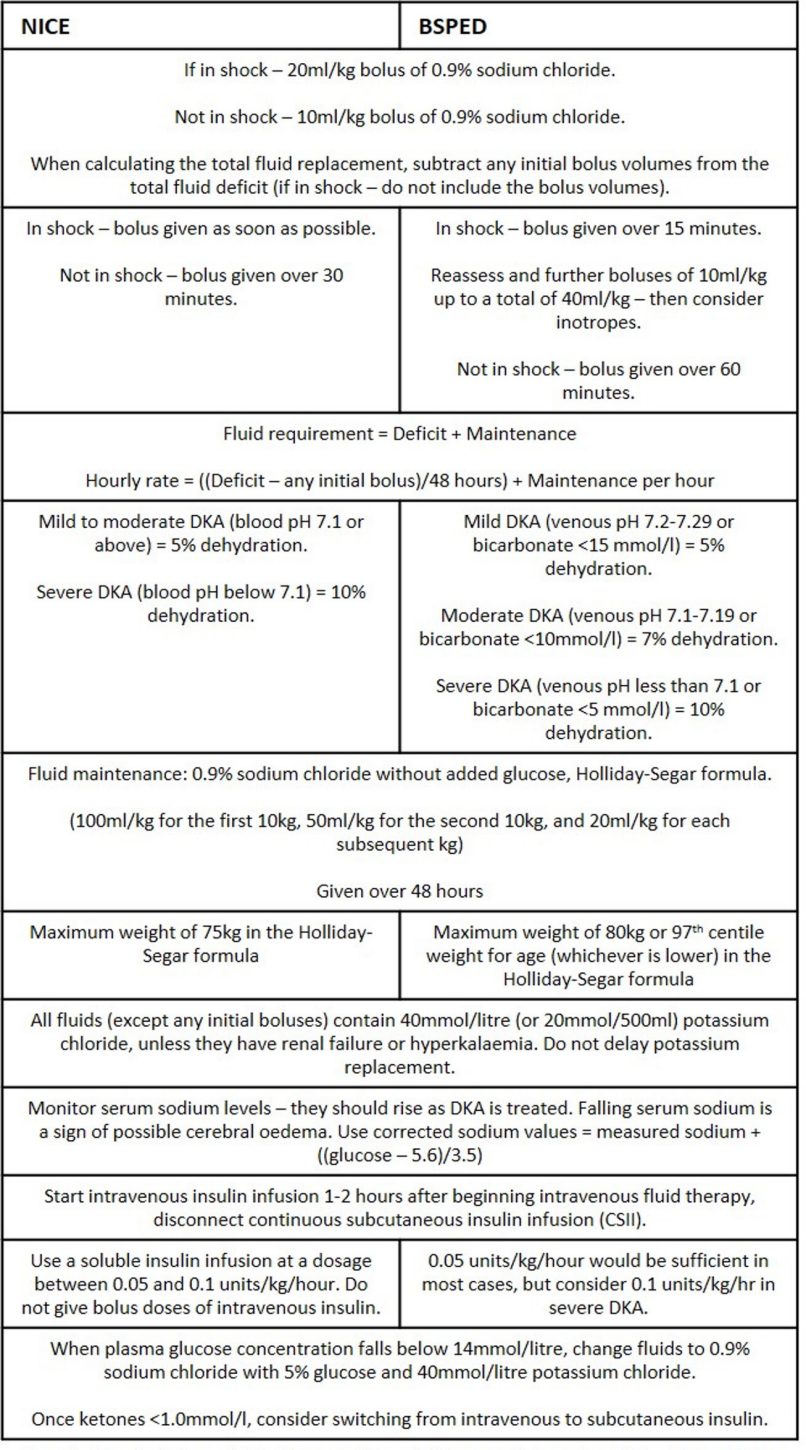
Flowchart demonstrating the similarities and differences between the 2020 updated guidelines. BSPED, British Society for Paediatric Endocrinology and Diabetes; DKA, diabetic ketoacidosis; NICE, National Institute for Health and Care Excellence.

Figure 2 highlights the key similarities and differences between the updated 2020 guidelines.

## Comparison to international guidance

The new guidelines released by both NICE^7^ and BSPED^6^ read similarly to those of non-UK-based origin. The 2018 consensus guidelines released by ISPAD^5^ in conjunction with the European Society for Paediatric Endocrinology and the Lawson Wilkins Paediatric Endocrine Society are used globally as standard guidelines for paediatric DKA management. Although the updated NICE and BSPED guidelines are very similar, as reviewed in figure 2, the BSPED guidelines appear to be most similar to the ISPAD guidelines on fluid resuscitation and electrolyte balance, with very few differences between the two.

The most pertinent similarities between the BSPED and ISPAD guidelines include giving a bolus for shock (20 mL/kg), using the Holliday-Segar formula to determine the maintenance fluid requirements, using a maximum of 80 kg in weight for these calculations and stratifying the severity of DKA (into mild, moderate and severe) compared with the two classifications used by NICE (mild/moderate and severe).

In contrast to the NICE and BSPED guidelines, which state that a 0.9% sodium chloride solution must be used when considering fluid requirements, in the ISPAD guidance, either a 0.45% or 0.9% sodium chloride or a balanced salt solution (Ringer’s lactate, Hartmann’s solution or Plasma-Lyte) may be used. This is secondary to recent findings from randomised control trials showing no difference in cerebral injury in patients rehydrated at different rates with either 0.45% or 0.9% sodium chloride. Additionally, for those not in shock, these fluids should be infused over 30–60 min, which falls between the two 2020 guidelines (NICE states to give fluids over 30 min and BSPED states over 60 min). Regarding the fluid bolus for those in shock, as with the NICE 2020 guidelines, the ISPAD guidelines state that it should be given ‘as soon as possible’. Conversely, BSPED says this should be over 15 min. However, in practice, this difference in nomenclature is likely to be negligible. Also, in agreement with the NICE 2020 guidelines, the ISPAD guidelines recommend the prescription of insulin at between 0.05 units/kg/hour and 0.1 units/kg/hour, where 0.05 units/kg/hour is usually sufficient in mild DKA. When assessing glucose introduction, the ISPAD guidance recommends that healthcare professionals ‘consider adding glucose before 17 mmol/L if falling rapidly. Otherwise 5% glucose should be added when the plasma glucose falls to approximately 14–17 mmol/L’. This is in contrast to the NICE and BSPED guidelines, which only introduce 5% glucose when it has fallen below 14 mmol/L.

The Royal Children’s Hospital (RCH) in Melbourne, Clinical Practice Guidelines for Diabetic Ketoacidosis, last updated in November 2018, have been adapted for statewide use with the support of the Victorian Paediatric Clinical Network. Although broadly similar, there are a few distinct differences between the RCH guidelines and those of NICE, BSPED and ISPAD. For those in shock, a 10 mL/kg 0.9% sodium chloride bolus can be given, half the volume of 20 mL/kg recommended by the other guidelines. Although initial fluid rates are based on a degree of dehydration (with the RCH stratifications varying from the other guidelines: mild≤4%, moderate=4%–7% and severe≥7%), maintenance fluids are not calculated using the Holliday-Segar formula. Instead a detailed table is used, with the fluid requirements classified according to the different degrees of dehydration up to a maximum of 70 kg.

However, as with the other guidelines, 40 mmol/L of potassium chloride is added to the fluid, as long as the child is not anuric or a serum potassium ≥5.5 mmol/L. If required, these can be increased to a maximum of 60 mmol/L, which is not specified in the other guidelines. Additionally, 5% glucose is added if the blood glucose is ≤15 mmol/L, which is comparable, although this can be increased to 10% glucose if blood glucose is falling rapidly or ≤5 mmol/L. Insulin infusion rate of 0.1 units/kg/hour is recommended unless treating children under 5 years of age, children undergoing inter-hospital transfer (where limited access to biochemical monitoring) or children with blood glucose <15 mmol/L at the time of commencement of the insulin infusion. This is placed at the upper end of the bracket of the other guidelines, where 0.1 units/kg/hour is recommended for those with severe DKA, adolescence or unresolved ketosis.

## Conclusions and recommendations

The convergence of the NICE and BSPED guidelines is a welcome development in the emergency management of paediatric DKA. This will provide much needed clarity to the treating clinicians, reducing regional variation and decreasing the potential for error. Despite the pathophysiology of CO remaining uncertain, fluid therapy must be carefully controlled, as inadequate resuscitation is likely to increase the risk of brain injury and thus must be avoided.

There is emerging evidence for a more judicious approach in the management of DKA^9^ and the NICE and BSPED guidelines are now largely concordant in this regard. A number of minor differences in the guidelines remain, but these are unlikely to profoundly influence patient management or outcomes. As both guidelines have changed considerably since their conception, and now have only minor differences, it could be argued that there is no benefit of two guidelines. The existence of two guidelines in the UK is primarily historical, with both guideline-producing groups having good reason to be interested in this area. However, the UK would, in our opinion, be better served with a single set of guidance to avoid confusion and harmonise practice.

Despite this, it is imperative that the incidence of CO continues to be monitored following a change to the guidelines and, in addition, this may offer insights into the underlying pathophysiological basis and effective preventative measures.

In the meantime, for junior clinicians treating DKA in CYP, close supervision by experienced paediatricians, careful monitoring and adherence to a single-set of evidence-based guidelines are likely to translate into better outcomes for this challenging condition.

## Supplementary Material

Author's manuscript

## Data Availability

Data sharing not applicable as no datasets generated and/or analysed for this study. Not applicable.
