# Supplementary material for: Update and harmonisation of guidance for the management of diabetic ketoacidosis in children and young people in the UK
Source: BMJ Paediatr Open. 2021 Jun 4;5(1):e001079. doi: 10.1136/bmjpo-2021-001079 (PMC8183192; doi:10.1136/bmjpo-2021-001079)
Supplement: Author's manuscript [file bmjpo-2021-001079.draft_revisions.pdf]

BMJ Paediatrics Open

BMJ Paediatrics Open is committed to open peer review. As part of this commitment we make the peer review history of every article we publish publicly available.

When an article is published we post the peer reviewers' comments and the authors' responses online. We also post the versions of the paper that were used during peer review. These are the versions that the peer review comments apply to.

The versions of the paper that follow are the versions that were submitted during the peer review process. They are not the versions of record or the final published versions. They should not be cited or distributed as the published version of this manuscript.

BMJ Paediatrics Open is an open access journal and the full, final, typeset and author-corrected version of record of the manuscript is available on our site with no access controls, subscription charges or pay-per-view fees (http://bmjpaedsopen.bmj.com).

If you have any questions on BMJ Paediatrics Open's open peer review process please email info.bmjpo@bmj.com

BMJ Paediatrics Open

Update and harmonisation of guidance for the management of diabetic ketoacidosis in children and young people

Journal:	*BMJ Paediatrics Open*
Manuscript ID	bmjpo-2021-001079
Article Type:	Review
Date Submitted by the Author:	02-Mar-2021
Complete List of Authors:	Rugg-Gunn, Charlotte; University of Liverpool School of Medicine; University of Liverpool Department of Women's and Children's Health<br>Deakin, Mark; Alder Hey Children's Hospital<br>Hawcutt, Daniel B; University of Liverpool, Department of Women's and Children's Health; NIHR Alder Hey Clinical Research Facility
Keywords:	Resuscitation, Diabetes

SCHOLARONE™
Manuscripts

I, the Submitting Author has the right to grant and does grant on behalf of all authors of the Work (as defined in the below author licence), an exclusive licence and/or a non-exclusive licence for contributions from authors who are: i) UK Crown employees; ii) where BMJ has agreed a CC-BY licence shall apply, and/or iii) in accordance with the terms applicable for US Federal Government officers or employees acting as part of their official duties; on a worldwide, perpetual, irrevocable, royalty-free basis to BMJ Publishing Group Ltd ("BMJ") its licensees and where the relevant Journal is co-owned by BMJ to the co-owners of the Journal, to publish the Work in this journal and any other BMJ products and to exploit all rights, as set out in our licence.

The Submitting Author accepts and understands that any supply made under these terms is made by BMJ to the Submitting Author unless you are acting as an employee on behalf of your employer or a postgraduate student of an affiliated institution which is paying any applicable article publishing charge ("APC") for Open Access articles. Where the Submitting Author wishes to make the Work available on an Open Access basis (and intends to pay the relevant APC), the terms of reuse of such Open Access shall be governed by a Creative Commons licence – details of these licences and which Creative Commons licence will apply to this Work are set out in our licence referred to above.

Other than as permitted in any relevant BMJ Author's Self Archiving Policies, I confirm this Work has not been accepted for publication elsewhere, is not being considered for publication elsewhere and does not duplicate material already published. I confirm all authors consent to publication of this Work and authorise the granting of this licence.

Confidential: For Review Only

Update and harmonisation of guidance for the management of diabetic ketoacidosis in children and young people

Charlotte Rugg-Gunn[1,2], Mark Deakin[3], Daniel B Hawcutt[2,4]

1: University of Liverpool Medical School, Liverpool, UK

2: Department of Women's and Children's Health, University of Liverpool, Liverpool, UK

3: Alder Hey Children's Hospital, Eaton Road, Liverpool, UK

4: NIHR Alder Hey Clinical Research Facility, Eaton Road, UK

Word Count: 1650

Figures: 1

Tables: 1

Keywords: Paediatrics, Diabetic Ketoacidosis, Insulin, Resuscitation, Guideline, Diabetes

Confidential: For Review Only

ABSTRACT

Diabetic ketoacidosis (DKA) is a life-threatening complication of Type 1 Diabetes Mellitus. Careful and timely intervention is required to optimise glycaemic control and reduce the risk of mortality and devastating complications. Of these, cerebral oedema is the leading cause of death, with a mortality rate of approximately 25%. This article highlights the recent updates to fluid therapy guidelines for DKA and provides clinical context for the benefit of paediatricians and junior doctors in light of this new guidance.

KEY MESSAGES

- Discordance between 2015 NICE and BSPED guidelines on fluid management for DKA in children and young people raised concerns about increased risk of cerebral oedema.
- Updates to both guidelines in 2020 has improved concordance, with 0.9% sodium chloride and the Holliday-Segar formula used as gold-standard to calculate fluid maintenance requirement.
- Minor differences remain, such as the maximum weight for maintenance and the stratification of DKA or dehydration severity.
- Close supervision by seniors, careful monitoring and adherence to guidelines should contribute to a reduction in deaths secondary to cerebral oedema.
- Further research is required to evaluate the efficacy of these new guidelines, and better understand the pathophysiology and risk factors for developing cerebral oedema.

Confidential: For Review Only

INTRODUCTION

Diabetic ketoacidosis (DKA) is a potentially life-threatening metabolic complication of Type 1 Diabetes Mellitus (T1DM). Despite interventions to reduce the incidence of DKA, the National Paediatric Diabetes Audit (NPDA) for 2018/19 showed that overall 20.9% of all newly diagnosed patients in the United Kingdom (UK) presented in DKA, although there is considerable regional variability.(2)

DKA is characterised by uncontrolled hyperglycaemia, ketosis and subsequent metabolic acidosis. As highlighted in Figure 1, the clinical picture can develop insidiously due to a variable constellation of non-specific systemic signs and symptoms including polyuria, polydipsia, weight loss, fatigue, vomiting, and abdominal pain, ultimately leading to confusion, coma, and death if untreated (3, 4). Due to the ambiguity of the presenting clinical features in the early stages, delays in diagnosis are common.(5)

In the UK, management of paediatric DKA is guided by the integrated care pathways of either the British Society for Paediatric Endocrinology and Diabetes (BSPED) or the National Institute for Health and Care Excellence (NICE). Both guidelines cover the diagnosis and management of Type 1 and Type 2 diabetes in children and young people (CYP) aged under 18.(6) Areas of particular benefit within a secondary care setting are centred on the requirements for fluid therapy, given the importance of resuscitation in preventing complications. Due to the difference in perceived risk of cerebral oedema (CO) due to rapid fluid administration, there was discordance between the NICE and BSPED 2015 guidelines.

Specifically, the NICE 2015 guidelines were more conservative with fluid administration, in order to avoid rapid changes in osmolality. The BSPED guidelines permitted a more generous fluid allowance, and this raised concerns about precipitating CO. The conflicting advice generated challenges in the emergency setting, with arbitrary site-specific preference rather than evidence-based medicine taking precedence. In addition to generating uncertainty of treatment pathways increasing the risk of clinical error, the regional variability hampered service evaluation and audit of clinical practice.

Unfortunately, there is little data to establish their efficacy, especially surrounding the mortality rates of DKA in the paediatric population. Consequently, further research on a national scale is required to assess this.

UPDATED GUIDANCE

In January 2020, the BSPED published new guidance for the management of children with DKA.(1) This integrated care pathway migrated further from the model of restrictive fluid replacement towards an even more flexible approach for resuscitative and maintenance fluids. The rationale for this was based on new evidence, such as the PECARN DKA FLUID trial, which showed that the initial conscious level was closely related to pH and weakly to age, but not to blood glucose or plasma sodium level.(19, 20) Whilst this appears to show that cerebral function is related to the severity of acidosis even in the absence of drivers of cerebral oedema, it does not negate previous evidence that fluid shift may also contribute towards cerebral oedema.(11, 12)

Furthermore, there are clear risks associated with inadequate fluid replacement. A hypovolaemic state will result in systemic hypotension and ultimately, cerebral hypoperfusion which, particularly in the context of acidosis, will increase the risk of a brain injury.(1)

In 2008, following three deaths from cerebral oedema, the South Thames Retrieval Service introduced a “restrictive fluid” DKA guideline in the region. Subsequently, no deaths have been reported but this is insufficient evidence on which to make definitive recommendations or guidelines.(21)

Confidential: For Review Only

More recently, in December 2020, NICE released updated guidance on the management of DKA in CYP which was broadly concordant with BSPED, particularly with respect to less cautious fluid restriction. This was prompted by new evidence identified by NICE's surveillance team in a review of 12 studies set to determine the optimal route of administration, type of fluid (including additives) as well as rate and volume for rehydration in CYP with DKA. The fluid protocols followed in the PECARN study were fundamental to these updates,(19) as the NICE committee noted that "the trial highlighted that restrictions to fluid administration as advised in the 2015 guideline were not necessarily required."(22) Additionally, as with the BSPED and International Society for Paediatric and Adolescent Diabetes (ISPAD) guidelines, the updated NICE guidance was amended to include the Holliday-Segar formula, used to calculate fluid deficit.(1, 6) 2020 updates to both the BSPED and NICE guidelines have a more permissive fluid rate than in the previous DKA fluid therapy guidelines.

Both BSPED and NICE recommend the following fluid maintenance requirements, using 0.9% sodium chloride without added glucose, and the Holliday-Segar formula:

- 100ml/kg for the first 10kg (0-10kg body weight)
- 50ml/kg for the second 10kg (10-20kg body weight)
- 20ml/kg for each subsequent kg (>20kg body weight)
- When calculating the total fluid replacement, subtract any initial bolus volumes from the total fluid deficit (unless the child or young person is in shock)

For insulin and electrolyte replacement requirements, both recommend:(1, 6)

- The addition of 40mmol/l of potassium chloride to all fluids (apart from the initial IV bolus) unless the patient is anuric or hyperkalaemic. Include this before starting the insulin infusion if hypokalaemia is observed at presentation.
- 0.9% NaCl should be used without added glucose unless the plasma glucose is <14mmol/l.
- Monitor sodium levels throughout treatment and calculate sodium initially to identify if hyponatraemia is present. This should be treated as soon as blood glucose falls. It is important to monitor as hyponatraemia and rapidly increasing sodium levels can both be signs of CO.
- Do not give IV sodium bicarbonate to CYP with DKA unless they have compromised cardiac contractility caused by life-threatening hyperkalaemia or severe acidosis, and you have discussed with paediatric intensivist.
- A soluble insulin infusion of between 0.05 and 0.1 units/kg/hour, 1-2 hours after IV fluids have been commenced. BSPED stated that the 0.05 units/kg/hr should be sufficient unless the patient is suffering from severe DKA or is perhaps an adolescent. If they are attached to a pump, disconnect when starting IV insulin therapy. In patients already on long-acting insulin, it should be continued, and in new patients consider commencing long-acting subcutaneous insulin alongside IV.

Although these guidelines now have greater cohesion, a number of minor differences remain:(1, 6)

- Regarding fluid replacement, NICE have included 2 levels of severity of DKA, whilst BSPED have subdivided the stratification to include 3 discrete categories for severity and subsequent dehydration status, as outlined in Table 1.
- For patients who are clinically dehydrated, but not in shock, an IV bolus of normal saline of 10ml/kg is recommended on admission, and this should be administered over 30 minutes (NICE) or 60 minutes (BSPED). This volume is included in the fluid replacement when calculating the total fluid deficit. NICE recommend discussing with a responsible senior paediatrician before giving more than one IV bolus. A second bolus should only be considered if needed to improve tissue perfusion and this must be done after reassessing their clinical status.

Confidential: For Review Only

- In the presence of hypovolaemic shock both NICE and BSPED recommend an IV 0.9% saline bolus of 20ml/kg. BSPED recommend repeating these infusions over 15 minutes to a maximum of 40ml/kg, followed by inotropes. In both guidelines, the bolus for shock is not subtracted from total fluid deficit.
- When calculating fluid replacement, the NICE guidelines recommend a maximum weight of 75kg whereas BSPED recommend a maximum weight of 80kg. This discrepancy is unlikely to be encountered on a frequent basis in general paediatric practice, and it may be more appropriate for young people of this size to be managed according to adult guidelines.

CONCLUSIONS AND RECOMMENDATIONS:

The convergence of the NICE and BSPED guidelines is a welcome development in the emergency management of paediatric DKA. This will provide much needed clarity to the treating clinicians, reducing regional variation and decreasing the potential for error. Despite the pathophysiology of CO remaining uncertain, there is emerging evidence for a more judicious approach in the management of DKA(20) and the NICE and BSPED guidelines are now largely concordant in this regard. A number of minor differences in the guidelines remain but these are unlikely to profoundly influence patient management or outcomes. It is imperative that the incidence of CO continues to be monitored following a change to the guidelines and, in addition, this may offer insights into the underlying pathophysiological basis and effective preventative measures.

In the meantime, for junior clinicians treating DKA in CYP, close supervision by senior, experienced paediatricians, careful monitoring and adherence to a single-set of evidence-based guidelines are likely to translate into better outcomes for this challenging condition.

Additional learning points:

- Children who are alert, not clinically dehydrated and not nauseated or vomiting, do not always require IV fluids, even if their ketone levels are high.(1) They usually tolerate oral rehydration and subcutaneous insulin but still require regular monitoring.
- When evaluating a patient in DKA, establish a clear pre-existing history of diabetes including usual insulin regimen details and adherence.
- Always investigate the cause of DKA. Common precipitants include sepsis or intercurrent infection, or there may be adverse psychosocial factors.
- Aim to replace the deficit evenly over the first 48 hours, but in critically ill CYP, discuss the fluid regimen early with the senior paediatric or paediatric intensivist (or both) due to increased risk of CO.(6)
- Consider stopping IV fluid therapy for DKA in a CYP if: ketosis is resolving and their blood pH has reached 7.3 **and** they are alert **and** they can take oral fluids without nausea or vomiting. Discuss with responsible senior paediatrician before stopping.(6)
- For a CYP with DKA who is using insulin pump therapy, restart the pump at least 60 minutes before stopping IV insulin. In addition, change the insulin cartridge and infusion set and insert the cannula into a new subcutaneous site.(1) For those without a pump, start subcutaneous insulin at least 30 minutes before stopping IV insulin.(6)
- Cerebral oedema is unpredictable, occurs more frequently in younger children and newly diagnosed diabetes, and has a mortality of around 25.%.(1)
- Despite concerns over increased fluid resuscitation, inadequate resuscitation is likely to increase the risk of brain injury and thus must be avoided.

Confidential: For Review Only

Author contributorship: Concept MD, writing CRG, DH, MD, editing DH, MD

Competing interests and funding: none declared

I, the Submitting Author has the right to grant and does grant on behalf of all authors of the Work (as defined in the below author licence), an exclusive licence and/or a non-exclusive licence for contributions from authors who are: i) UK Crown employees; ii) where BMJ has agreed a CC-BY licence shall apply, and/or iii) in accordance with the terms applicable for US Federal Government officers or employees acting as part of their official duties; on a worldwide, perpetual, irrevocable, royalty-free basis to BMJ Publishing Group Ltd ("BMJ") its licensees and where the relevant Journal is co-owned by BMJ to the co-owners of the Journal, to publish the Work in BMJ Paediatrics Open and any other BMJ products and to exploit all rights, as set out in our licence.

The Submitting Author accepts and understands that any supply made under these terms is made by BMJ to the Submitting Author unless you are acting as an employee on behalf of your employer or a postgraduate student of an affiliated institution which is paying any applicable article publishing charge ("APC") for Open Access articles. Where the Submitting Author wishes to make the Work available on an Open Access basis (and intends to pay the relevant APC), the terms of reuse of such Open Access shall be governed by a Creative Commons licence – details of these licences and which Creative Commons licence will apply to this Work are set out in our licence referred to above.

References:

1. Integrated care pathway for the management of children and young people with Diabetic Ketoacidosis British Society for Paediatric Endocrinology and Diabetes; 2020 [Available from: https://www.bsped.org.uk/media/1742/dka-icp-2020-v1_1.pdf.
2. Schnabel A, Hedrich CM. Childhood Vasculitis. Frontiers in pediatrics. 2018;6:421.
3. Wolfsdorf J, Glaser N, Sperling MA. Diabetic Ketoacidosis in Infants, Children, and Adolescents: A consensus statement from the American Diabetes Association. Diabetes Care. 2006;29(5):1150-9.
4. Ribero S, Sciascia S, Borradori L, Lipsker D. The Cutaneous Spectrum of Lupus Erythematosus. Clinical reviews in allergy & immunology. 2017;53(3):291-305.
5. Unnikrishnan R, Shah VN, Mohan V. Challenges in diagnosis and management of diabetes in the young. Clinical Diabetes and Endocrinology. 2016;2(1).
6. Diabetes (type 1 and type 2) in children and young people: diagnosis and management: NICE guideline NG18; 2020 [Available from: https://pathways.nice.org.uk/pathways/diabetes-in-children-and-young-people/diabetic-ketoacidosis-in-children-and-young-people#content=view-node%3Anodes-recognition-referral-and-diagnosis.
7. Edge JA, Hawkins MM, Winter DL, Dunger DB. The risk and outcome of cerebral oedema developing during diabetic ketoacidosis. Arch Dis Child. 2001;85(1):16-22.
8. Glaser N, Barnett P, McCaslin I, Nelson D, Trainor J, Louie J, et al. Risk Factors for Cerebral Edema in Children with Diabetic Ketoacidosis. New England Journal of Medicine. 2001;344(4):264-9.
9. Lawrence SE, Cummings EA, Gaboury I, Daneman D. Population-based study of incidence and risk factors for cerebral edema in pediatric diabetic ketoacidosis. J Pediatr. 2005;146(5):688-92.
10. Glaser NS, Wootton-Gorges SL, Buonocore MH, Marcin JP, Rewers A, Strain J, et al. Frequency of sub-clinical cerebral edema in children with diabetic ketoacidosis. Pediatr Diabetes. 2006;7(2):75-80.
11. Duck SC, Wyatt DT. Factors associated with brain herniation in the treatment of diabetic ketoacidosis. J Pediatr. 1988;113(1 Pt 1):10-4.
12. Arieff AI, Kleeman CR. Studies on mechanisms of cerebral edema in diabetic comas. Effects of hyperglycemia and rapid lowering of plasma glucose in normal rabbits. J Clin Invest. 1973;52(3):571-83.
13. Arieff AI, Kleeman CR. Cerebral edema in diabetic comas. II. Effects of hyperosmolality, hyperglycemia and insulin in diabetic rabbits. J Clin Endocrinol Metab. 1974;38(6):1057-67.
14. Prockop LD. Hyperglycemia, Polyol Accumulation, and Increased Intracranial Pressure. Archives of Neurology. 1971;25(2):126-40.
15. Harris GD, Fiordalisi I, Finberg L. Safe management of diabetic ketoacidemia. J Pediatr. 1988;113(1 Pt 1):65-8.
16. Kamel KS, Halperin ML. Acid-base problems in diabetic ketoacidosis. N Engl J Med. 2015;372(6):546-54.

Confidential: For Review Only

17. Glaser N. Cerebral injury and cerebral edema in children with diabetic ketoacidosis: could cerebral ischemia and reperfusion injury be involved? Pediatr Diabetes. 2009;10(8):534-41.
18. L. Rosenbloom A. Prevention of Type 1 Diabetes: Perspectives. The Journal of Clinical Endocrinology & Metabolism. 2000;85(2):494-5.
19. Kuppermann N, Ghetti S, Schunk JE, Stoner MJ, Rewers A, McManemy JK, et al. Clinical Trial of Fluid Infusion Rates for Pediatric Diabetic Ketoacidosis. N Engl J Med. 2018;378(24):2275-87.
20. Edge JA, Roy Y, Bergomi A, Murphy NP, Ford-Adams ME, Ong KK, et al. Conscious level in children with diabetic ketoacidosis is related to severity of acidosis and not to blood glucose concentration. Pediatr Diabetes. 2006;7(1):11-5.
21. Lillie J, Boot E, Tibby SM, McDougall M, Nyman A, Griffiths B, et al. Management of fluids in paediatric diabetes ketoacidosis: concerns over new guidance. Arch Dis Child. October 2020;105:1019-20.
22. Diabetes (type 1 and type 2) in children and young people: diagnosis and management. Evidence reviews for fluid therapy for the management of diabetic ketoacidosis. Guideline version (Draft). National Institute for Health and Care Excellence; 2020.

Clinical history:

- Polyuria/polydipsia
- Weight loss
- Abdominal pain
- Weakness
- Vomiting
- Confusion

Clinical signs:

- Dehydration
- Kussmaul breathing
- Ketotic smell
- Lethargy, drowsiness

Biochemistry:

- Hyperglycaemia (>11mmol/L)
- Acidaemia (pH<7.3)
- Ketosis (blood ketones >3mmol/L or urine ketones ++)

Potential triggers:

- Infection e.g. viral
- Interruption to insulin therapy e.g. pump malfunction or omitted doses
- Physical or emotional trauma
- Pregnancy
- Drug interactions including alcohol and particularly cocaine

Figure 1: Key signs and symptoms of Diabetic Ketoacidosis, adapted from(1)

Confidential: For Review Only

Guidelines	Severity of DKA	Dehydration
NICE	Mild-moderate – blood pH ≥7.1<br>Severe – blood pH <7.1	5%<br>10%
BSPED	Mild - (venous pH 7.2-7.29 or bicarbonate <15mmol/l)<br>Moderate - (venous pH 7.1-7.19 or bicarbonate <10mmol/l)<br>Severe - (venous pH <7.1 or bicarbonate <5mmol/l)	5%<br>7%<br>10%

Table 1: comparison of NICE and BSPED DKA classification and dehydration status

Confidential: For Review Only

BMJ Paediatrics Open

Update and harmonisation of guidance for the management of diabetic ketoacidosis in children and young people in the UK

Journal:	*BMJ Paediatrics Open*
Manuscript ID	bmjpo-2021-001079.R1
Article Type:	Review
Date Submitted by the Author:	21-Apr-2021
Complete List of Authors:	Rugg-Gunn, Charlotte; University of Liverpool School of Medicine; University of Liverpool Department of Women's and Children's Health<br>Deakin, Mark; Alder Hey Children's Hospital<br>Hawcutt, Daniel B; University of Liverpool, Department of Women's and Children's Health; NIHR Alder Hey Clinical Research Facility
Keywords:	Resuscitation, Diabetes

SCHOLARONE™
Manuscripts

I, the Submitting Author has the right to grant and does grant on behalf of all authors of the Work (as defined in the below author licence), an exclusive licence and/or a non-exclusive licence for contributions from authors who are: i) UK Crown employees; ii) where BMJ has agreed a CC-BY licence shall apply, and/or iii) in accordance with the terms applicable for US Federal Government officers or employees acting as part of their official duties; on a worldwide, perpetual, irrevocable, royalty-free basis to BMJ Publishing Group Ltd ("BMJ") its licensees and where the relevant Journal is co-owned by BMJ to the co-owners of the Journal, to publish the Work in this journal and any other BMJ products and to exploit all rights, as set out in our licence.

The Submitting Author accepts and understands that any supply made under these terms is made by BMJ to the Submitting Author unless you are acting as an employee on behalf of your employer or a postgraduate student of an affiliated institution which is paying any applicable article publishing charge ("APC") for Open Access articles. Where the Submitting Author wishes to make the Work available on an Open Access basis (and intends to pay the relevant APC), the terms of reuse of such Open Access shall be governed by a Creative Commons licence – details of these licences and which Creative Commons licence will apply to this Work are set out in our licence referred to above.

Other than as permitted in any relevant BMJ Author's Self Archiving Policies, I confirm this Work has not been accepted for publication elsewhere, is not being considered for publication elsewhere and does not duplicate material already published. I confirm all authors consent to publication of this Work and authorise the granting of this licence.

Confidential: For Review Only

Update and harmonisation of guidance for the management of diabetic ketoacidosis in children and young people in the UK

Charlotte EM Rugg-Gunn[1,2], Mark Deakin[3], Daniel B Hawcutt[2,4]

1: University of Liverpool Medical School, Liverpool, UK

2: Department of Women's and Children's Health, University of Liverpool, Liverpool, UK

3: Alder Hey Children's Hospital, Eaton Road, Liverpool, UK

4: NIHR Alder Hey Clinical Research Facility, Eaton Road, UK

Word Count: 2881

Figures: 2

Tables: 1

Keywords: Paediatrics, Diabetic Ketoacidosis, Insulin, Resuscitation, Guideline, Diabetes

Confidential: For Review Only

ABSTRACT

Diabetic ketoacidosis (DKA) is a life-threatening complication of Type 1 Diabetes Mellitus. Careful and timely intervention is required to optimise glycaemic control and reduce the risk of mortality and devastating complications. Of these, cerebral oedema is the leading cause of death, with a mortality rate of approximately 25%. This article highlights the recent updates to fluid therapy guidelines for DKA and provides clinical context for the benefit of paediatricians and junior doctors in light of this new guidance.

KEY MESSAGES

- Updates to both guidelines in 2020 has improved concordance, with 0.9% sodium chloride and the Holliday-Segar formula used as gold-standard to calculate fluid maintenance requirement.
- Minor differences remain, such as the maximum weight for maintenance and the stratification of DKA or dehydration severity.
- Careful monitoring and adherence to these national guidelines is recommended and will hopefully contribute to a reduction in deaths secondary to cerebral oedema.
- Further research is required to evaluate the efficacy of these new guidelines, and better understand the pathophysiology and risk factors for developing cerebral oedema.

Confidential: For Review Only

INTRODUCTION

Diabetic ketoacidosis (DKA) is a potentially life-threatening metabolic complication of Type 1 Diabetes Mellitus (T1DM). Despite interventions to reduce the incidence of DKA, the National Paediatric Diabetes Audit (NPDA) for 2018/19 showed that overall 20.9% of all newly diagnosed patients in the United Kingdom (UK) presented in DKA, although there is considerable regional variability.(1)

DKA is characterised by uncontrolled hyperglycaemia, ketosis and subsequent metabolic acidosis. As highlighted in Figure 1, the clinical picture can develop insidiously due to a variable constellation of non-specific systemic signs and symptoms including polyuria, polydipsia, weight loss, fatigue, vomiting, and abdominal pain, ultimately leading to confusion, coma, and death if untreated (2, 3). Due to the ambiguity of the presenting clinical features in the early stages, delays in diagnosis are common.(4)

Globally, the International Society for Pediatric and Adolescent Diabetes (ISPAD) guidelines are widely used for management of paediatric DKA.(5) However, in the UK it is guided by the integrated care pathways of either the British Society for Paediatric Endocrinology and Diabetes (BSPED) or the National Institute for Health and Care Excellence (NICE). The BSPED guideline evolved from the NICE guideline in 2009 due to concerns over limited evidence that was used for its development. Whilst there were a number of dual-serving board members, the input and additional evidence by the BSPED special interest group in DKA was thought to increase its safety.(6) Both guidelines cover the diagnosis and management of Type 1 and Type 2 diabetes in children and young people (CYP) aged under 18.(7) Areas of particular benefit within a secondary care setting are centred on the requirements for fluid therapy, given the importance of resuscitation in preventing complications. Due to the difference in perceived risk of cerebral oedema (CO) due to rapid fluid administration, there was discordance between the NICE and BSPED 2015 guidelines.

Specifically, the NICE 2015 guidelines were more conservative with fluid administration, in order to avoid rapid changes in osmolality. The BSPED guidelines permitted a more generous fluid allowance, and this raised concerns about precipitating CO. The conflicting advice generated challenges in the emergency setting, with arbitrary site-specific preference rather than evidence-based medicine taking precedence. In addition to generating uncertainty of treatment pathways increasing the risk of clinical error, the regional variability hampered service evaluation and audit of clinical practice.

Unfortunately, there is little data to establish their efficacy, especially surrounding the mortality rates of DKA in the paediatric population. However, inadequate resuscitation is likely to increase the risk of brain injury and thus must be avoided. Further research on a national scale is required to assess this.

UPDATED GUIDANCE

In January 2020, the BSPED published new guidance for the management of children with DKA.(6) This integrated care pathway migrated further from the model of restrictive fluid replacement towards an even more flexible approach for resuscitative and maintenance fluids. The rationale for this was based on new evidence from the PECARN DKA Fluid trial, which suggested that there was no significant difference in the groups of rapid and slower fluid administration.(8) Additionally, it showed that the initial conscious level was closely related to pH and weakly to age, but not to blood glucose or plasma sodium level.(8, 9) Whilst this appears to demonstrate that cerebral function is related to the severity of acidosis even in the absence of drivers of cerebral oedema, it does not negate previous evidence that fluid shift may also contribute towards cerebral oedema.(10, 11)

Furthermore, there are clear risks associated with inadequate fluid replacement. A hypovolaemic state will result in systemic hypotension and ultimately, cerebral hypoperfusion which, particularly in the context of acidosis, will increase the risk of a brain injury.(6)

Confidential: For Review Only

In 2008, following three deaths from cerebral oedema, the South Thames Retrieval Service introduced a "restrictive fluid" DKA guideline in the region. Subsequently, no deaths have been reported but this is insufficient evidence on which to make definitive recommendations or guidelines.(12)

More recently, in December 2020, NICE released updated guidance on the management of DKA in CYP which was broadly concordant with BSPED, particularly with respect to less cautious fluid restriction. This was prompted by new evidence identified by NICE's surveillance team in a review of 12 studies set to determine optimal fluid therapy in CYP with DKA. The fluid protocols followed in the PECARN study were fundamental to these updates,(8) as "neither the rate of administration nor the sodium chloride content of intravenous fluids significantly influenced neurologic outcomes in children with diabetic ketoacidosis".(8) Therefore, restrictions to the fluid administration which were recommended in the 2015 NICE guidance "were not necessarily required".(13) Additionally, as with the BSPED and ISPAD guidelines, the updated NICE guidance was amended to include the Holliday-Segar formula, used to calculate fluid maintenance.(6, 7) 2020 updates to both the BSPED and NICE guidelines have a more permissive fluid rate than in the previous DKA fluid therapy guidelines.

Both BSPED and NICE recommend the following fluid maintenance requirements, using 0.9% sodium chloride without added glucose, and the Holliday-Segar formula:

- 100ml/kg for the first 10kg (0-10kg body weight)
- 50ml/kg for the second 10kg (10-20kg body weight)
- 20ml/kg for each subsequent kg (>20kg body weight)
- When calculating the total fluid replacement, subtract any initial bolus volumes from the total fluid deficit (unless the child or young person is in shock)

For insulin and electrolyte replacement requirements, both recommend:(6, 7)

- The addition of 40mmol/l potassium chloride to all fluids (apart from the initial IV bolus) unless the patient is anuric or hyperkalaemic. Include this before starting the insulin infusion if hypokalaemia is observed at presentation.
- 0.9% sodium chloride should be used without added glucose unless the plasma glucose is <14mmol/l.
- Monitor sodium levels throughout treatment and calculate sodium initially to identify if hyponatraemia is present. This should be treated as soon as blood glucose falls. It is important to monitor as hyponatraemia and rapidly increasing sodium levels can both be signs of CO.
- Do not give IV sodium bicarbonate to CYP with DKA unless they have compromised cardiac contractility caused by life-threatening hyperkalaemia or severe acidosis, and you have discussed with paediatric intensivist.
- A soluble insulin infusion of between 0.05 and 0.1 units/kg/hour, 1-2 hours after IV fluids have been commenced. BSPED stated that the 0.05 units/kg/hr should be sufficient unless the patient is suffering from severe DKA or is an adolescent. If they are attached to a 'continuous subcutaneous insulin infusion', disconnect when starting IV insulin therapy. For patients already on long-acting insulin, it may be continued, and in new patients consider commencing long-acting subcutaneous insulin alongside IV. Similarly, the NICE guidelines state that continuing subcutaneous basal insulin in a child or young person who was using a basal insulin before DKA may be continued in discussion with a diabetes specialist.

Although these guidelines now have greater cohesion, a number of minor differences remain:(1, 6)

- Regarding fluid replacement, NICE have included 2 levels of severity of DKA, whilst BSPED have subdivided the stratification to include 3 discrete categories for severity and subsequent dehydration status, as outlined in Table 1.
    - This remains a challenge due to the absence of continuity across the two guidelines. The impact could be great due to the difference between 5% dehydration and 7% dehydration when calculating fluid deficit, and thus risk the development of cerebral oedema. Given that the maximum weight

Confidential: For Review Only

considered for each is 75kg for NICE and 80kg for BSPED, the maximum difference in deficit fluids could be 1,850ml (5% x 75kg = 3750ml for NICE compared to 7% x 80kg = 5600ml). However, the difference only affects those with "moderate DKA" category as those with mild DKA are assumed to have 5% dehydration and severe DKA 10% dehydration in both guidelines.

- For patients who are clinically dehydrated, but not in shock, an IV 10 ml/kg bolus of 0.9% sodium chloride is recommended on admission. This should be administered over 30 minutes (NICE) or 60 minutes (BSPED). This volume is included in the fluid replacement when calculating the total fluid deficit. NICE recommend discussing with a paediatrician experienced in DKA management before giving more than one IV bolus. A second bolus should only be considered if needed to improve tissue perfusion and this must be done after reassessing their clinical status.
    - Although the bolus provided is identical, administering it over double the period of time recommended by NICE (in the case of the BSPED guidelines) could increase the risk of cerebral oedema secondary to prolonged dehydration. Given that there is a lack of clear evidence about whether the risk of CO is increased secondary to greater fluid administration, it is probable that they are being cautious. However, it must be ensured that this does not result in inadequate fluid resuscitation, which could further increase the risk of CO. Guidance from the ISPAD consensus released in 2018 states that this should be administered between 30-60 minutes which falls between the two UK guidelines.(5) Therefore, aiming to administer the fluid rapidly but observing for signs of potential deterioration and adjusting the rate accordingly will provide the best outcome for patients.
- In the presence of hypovolaemic shock both NICE and BSPED recommend an IV 0.9% sodium chloride bolus of 20ml/kg. BSPED recommend repeating these infusions over 15 minutes to a maximum of 40ml/kg, before considering the introduction of inotropes, whilst NICE recommends administering it as soon as possible. In both guidelines, the bolus for shock is not subtracted from total fluid deficit.
    - Whilst the nomenclature varies between these two guidelines, in practice it is likely that these will represent similar time frames. Administering a 20ml/kg 0.9% sodium chloride bolus to treat shock as quickly as possible should provide the best therapy for the patient.
- When calculating fluid replacement, the NICE guidelines recommend a maximum weight of 75kg whereas BSPED recommend a maximum weight of 80kg or 97th centile weight for age (whichever is lower).
    - This discrepancy is unlikely to be encountered on a frequent basis in general paediatric practice. However, if the weight greatly exceeds 75kg it may be more appropriate for young people to be managed according to adult guidelines due to their increased body habitus. Additionally, identifying a CYP on the 97th centile can be difficult, especially in an emergency setting, and therefore this may be missed.

Figure 2 highlights the key similarities and differences between the updated 2020 guidelines.

COMPARISON TO INTERNATIONAL GUIDANCE:

The new guidelines released by both NICE(7) and BSPED(6) read similarly to those of non-UK based origin. The 2018 consensus guidelines released by ISPAD(5) in conjunction with the European Society for Paediatric Endocrinology and the Lawson Wilkins Pediatric Endocrine Society (ESPE/LWPES) are used globally as standard guidelines for paediatric DKA management. Although the updated NICE and BSPED guidelines are very similar, as reviewed in Figure 2, the BSPED guidelines appear to be most similar to the ISPAD guidelines on fluid resuscitation and electrolyte balance, with very few differences between the two.

The most pertinent similarities between the BSPED and ISPAD guidelines include giving a bolus for shock (20mL/kg), using the Holliday-Segar formula to determine the maintenance fluid requirements, using a maximum of 80kg in weight for these calculations and stratifying the severity of DKA (into mild, moderate and severe) compared to the two classifications used by NICE (mild/moderate and severe).

Confidential: For Review Only

In contrast to the NICE and BSPED guidelines which state a 0.9% sodium chloride solution must be used when considering fluid requirements, in the ISPAD guidance, either a 0.45% or 0.9% sodium chloride or a balanced salt solution (Ringer's lactate, Hartmann's solution or PlasmaLyte) may be used. This is secondary to recent findings from randomised control trials (RCTs) showing no difference in cerebral injury in patients rehydrated at different rates with either 0.45% or 0.9% sodium chloride. Additionally, for those not in shock, these fluids should be infused over 30 to 60 minutes which falls between the two 2020 guidelines (NICE states to give fluids over 30 minutes, BSPED states over 60 minutes). Regarding the fluid bolus for those in shock, as with the NICE 2020 guidelines, the ISPAD guidelines state that it should be given "as soon as possible". Conversely, BSPED says this should be over 15 minutes. However, in practice this difference in nomenclature is likely to be negligible. Also in agreement with the NICE 2020 guidelines, the ISPAD guidelines recommend the prescription of insulin at between 0.05 and 0.1 units/kg/hour, where 0.05 units/kg/hour is usually sufficient in mild DKA. When assessing glucose introduction, the ISPAD guidance recommends that healthcare professionals "consider adding glucose before 17 mmol if falling rapidly. Otherwise 5% glucose should be added when the plasma glucose falls to approximately 14 to 17 mmol/L/". This is in contrast to the NICE and BSPED guidelines which only introduce 5% glucose when it has fallen below 14mmol/L.

The Royal Children's Hospital in Melbourne (RCH) Clinical Practice Guidelines for Diabetic Ketoacidosis, last updated in November 2018, have been adapted for statewide use with the support of the Victorian Paediatric Clinical Network. Although broadly similar, there are a few distinct differences between the RCH guidelines and those of NICE, BSPED and ISPAD. For those in shock, a 10mL/kg 0.9% sodium chloride bolus can be given, half the volume of 20mL/kg recommended by the other guidelines. Although initial fluid rates are based on a degree of dehydration (with the RCH stratifications varying from the other guidelines – mild = <4%, moderate = 4-7% and severe = >7%), maintenance fluids are not calculated using the Holliday-Segar formula. Instead a detailed table is used, with the fluid requirements classified according to the different degrees of dehydration up to a maximum of 70kg.

However, as with the other guidelines, 40mmol/L of potassium chloride is added to the fluid, as long as the child is not anuric or a serum potassium ≥5.5 mmol/L. If required, these can be increased to a maximum of 60mmol/L, which is not specified in the other guidelines. Additionally, 5% glucose is added if the blood glucose is ≤15 mmol/L, which is comparable, although this can be increased to 10% glucose if blood glucose is falling rapidly or ≤5 mmol/L. Insulin infusion rate of 0.1 units/kg/hour is recommended unless treating children under 5 years, children undergoing inter-hospital transfer (where limited access to biochemical monitoring) or children with blood glucose <15 mmol/L at the time of commencement of the insulin infusion. This is placed at the upper end of the bracket of the other guidelines, where 0.1 units/kg/hour is recommended for those with severe DKA, adolescence or unresolved ketosis.

CONCLUSIONS AND RECOMMENDATIONS:

The convergence of the NICE and BSPED guidelines is a welcome development in the emergency management of paediatric DKA. This will provide much needed clarity to the treating clinicians, reducing regional variation and decreasing the potential for error. Despite the pathophysiology of CO remaining uncertain, fluid therapy must be carefully controlled, as inadequate resuscitation is likely to increase the risk of brain injury and thus must be avoided.

There is emerging evidence for a more judicious approach in the management of DKA(9) and the NICE and BSPED guidelines are now largely concordant in this regard. A number of minor differences in the guidelines remain but these are unlikely to profoundly influence patient management or outcomes. As both guidelines have changed considerably since their conception, and now have only minor differences, it could be argued that there is no benefit of two guidelines. However, it is imperative that the incidence of CO continues to be monitored following a change to the guidelines and, in addition, this may offer insights into the underlying pathophysiological basis and effective preventative measures.

In the meantime, for junior clinicians treating DKA in CYP, close supervision by experienced paediatricians, careful monitoring and adherence to a single-set of evidence-based guidelines are likely to translate into better outcomes for this challenging condition.

Author contributorship: Concept MD, writing CRG, DH, MD, editing DH, MD

Competing interests and funding: none declared

I, the Submitting Author has the right to grant and does grant on behalf of all authors of the Work (as defined in the below author licence), an exclusive licence and/or a non-exclusive licence for contributions from authors who are: i) UK Crown employees; ii) where BMJ has agreed a CC-BY licence shall apply, and/or iii) in accordance with the terms applicable for US Federal Government officers or employees acting as part of their official duties; on a worldwide, perpetual, irrevocable, royalty-free basis to BMJ Publishing Group Ltd ("BMJ") its licensees and where the relevant Journal is co-owned by BMJ to the co-owners of the Journal, to publish the Work in BMJ Paediatrics Open and any other BMJ products and to exploit all rights, as set out in our licence.

The Submitting Author accepts and understands that any supply made under these terms is made by BMJ to the Submitting Author unless you are acting as an employee on behalf of your employer or a postgraduate student of an affiliated institution which is paying any applicable article publishing charge ("APC") for Open Access articles. Where the Submitting Author wishes to make the Work available on an Open Access basis (and intends to pay the relevant APC), the terms of reuse of such Open Access shall be governed by a Creative Commons licence – details of these licences and which Creative Commons licence will apply to this Work are set out in our licence referred to above.

References:

1. Schnabel A, Hedrich CM. Childhood Vasculitis. Frontiers in pediatrics. 2018;6:421.
2. Wolfsdorf J, Glaser N, Sperling MA. Diabetic Ketoacidosis in Infants, Children, and Adolescents: A consensus statement from the American Diabetes Association. Diabetes Care. 2006;29(5):1150-9.
3. Ribero S, Sciascia S, Borradori L, Lipsker D. The Cutaneous Spectrum of Lupus Erythematosus. Clinical reviews in allergy & immunology. 2017;53(3):291-305.
4. Unnikrishnan R, Shah VN, Mohan V. Challenges in diagnosis and management of diabetes in the young. Clinical Diabetes and Endocrinology. 2016;2(1).
5. Wolfsdorf JI, Glaser N, Agus M, Fritsch M, Hanas R, Rewers A, et al. ISPAD Clinical Practice Consensus Guidelines 2018: Diabetic ketoacidosis and the hyperglycemic hyperosmolar state. Pediatr Diabetes. 2018;19 Suppl 27:155-77.
6. Integrated care pathway for the management of children and young people with Diabetic Ketoacidosis British Society for Paediatric Endocrinology and Diabetes; 2020 [Available from: https://www.bsped.org.uk/media/1742/dka-icp-2020-v1_1.pdf.
7. Diabetes (type 1 and type 2) in children and young people: diagnosis and management: NICE guideline NG18; 2020 [Available from: https://pathways.nice.org.uk/pathways/diabetes-in-children-and-young-people/diabetic-ketoacidosis-in-children-and-young-people#content=view-node%3Anodes-recognition-referral-and-diagnosis.
8. Kuppermann N, Ghetti S, Schunk JE, Stoner MJ, Rewers A, McManemy JK, et al. Clinical Trial of Fluid Infusion Rates for Pediatric Diabetic Ketoacidosis. N Engl J Med. 2018;378(24):2275-87.
9. Edge JA, Roy Y, Bergomi A, Murphy NP, Ford-Adams ME, Ong KK, et al. Conscious level in children with diabetic ketoacidosis is related to severity of acidosis and not to blood glucose concentration. Pediatr Diabetes. 2006;7(1):11-5.
10. Duck SC, Wyatt DT. Factors associated with brain herniation in the treatment of diabetic ketoacidosis. J Pediatr. 1988;113(1 Pt 1):10-4.
11. Arieff AI, Kleeman CR. Studies on mechanisms of cerebral edema in diabetic comas. Effects of hyperglycemia and rapid lowering of plasma glucose in normal rabbits. J Clin Invest. 1973;52(3):571-83.

For peer review only

12. Lillie J, Boot E, Tibby SM, McDougall M, Nyman A, Griffiths B, et al. Management of fluids in paediatric diabetes ketoacidosis: concerns over new guidance. Arch Dis Child. October 2020;105:1019-20.
13. Diabetes (type 1 and type 2) in children and young people: diagnosis and management. Evidence reviews for fluid therapy for the management of diabetic ketoacidosis. Guideline version (Draft). National Institute for Health and Care Excellence; 2020.

Confidential: For Review Only

Clinical history:

- Polyuria/polydipsia
- Weight loss
- Abdominal pain
- Weakness
- Vomiting
- Confusion

Clinical signs:

- Dehydration
- Kussmaul breathing
- Ketotic smell
- Lethargy, drowsiness

Biochemistry:

- Hyperglycaemia (>11mmol/L)
- Acidaemia (pH<7.3)
- Ketosis (blood ketones >3mmol/L or urine ketones ++)

Potential triggers:

- Infection e.g. viral
- Interruption to insulin therapy e.g. pump malfunction or omitted doses
- Physical or emotional trauma
- Pregnancy
- Drug interactions including alcohol and particularly cocaine

Figure 1: Key signs and symptoms of Diabetic Ketoacidosis, adapted from (1)

64x137mm (150 x 150 DPI)

<table>
<tr><th>NICE</th><th>BSPED</th></tr>
<tr><td colspan="2">If in shock - 20ml/kg bolus of 0.9% sodium chloride.

Not in shock – 10ml/kg bolus of 0.9% sodium chloride.

When calculating the total fluid replacement, subtract any initial bolus volumes from the total fluid deficit (if in shock – do not include the bolus volumes).</td></tr>
<tr><td>In shock – bolus given as soon as possible.

Not in shock – bolus given over 30 minutes.</td><td>In shock – bolus given over 15 minutes.

Reassess and further boluses of 10ml/kg up to a total of 40ml/kg – then consider inotropes.

Not in shock - bolus given over 60 minutes.</td></tr>
<tr><td colspan="2">Fluid requirement = Deficit + Maintenance

Hourly rate = ((Deficit – any initial bolus) / 48 hours) + Maintenance per hour</td></tr>
<tr><td>Mild to moderate DKA (blood pH 7.1 or above) = 5% dehydration.

Severe DKA (blood pH below 7.1) = 10% dehydration.</td><td>Mild DKA (venous pH 7.2-7.29 or bicarbonate <15 mmol/l) = 5% dehydration.

Moderate DKA (venous pH 7.1-7.19 or bicarbonate <10mmol/l) = 7% dehydration.

Severe DKA (venous pH less than 7.1 or bicarbonate <5 mmol/l) = 10% dehydration.</td></tr>
<tr><td colspan="2">Fluid maintenance: 0.9% sodium chloride without added glucose, Holliday-Segar formula.

(100mg/kg for the first 10kg, 50ml/kg for the second 10kg, and 20ml/kg for each subsequent kg)

Given over 48 hours</td></tr>
<tr><td>Maximum weight of 75kg in the Holliday-Segar formula</td><td>Maximum weight of 80kg or 97th centile weight for age (whichever is lower) in the Holliday-Segar formula</td></tr>
<tr><td colspan="2">All fluids (except any initial boluses) contain 40mmol/litre (or 20mmol/500ml) potassium chloride, unless they have renal failure or hyperkalaemia. Do not delay potassium replacement.</td></tr>
<tr><td colspan="2">Monitor serum sodium levels - they should rise as DKA is treated. Falling serum sodium is a sign of possible cerebral oedema. Use corrected sodium values = measured sodium + ((glucose-5.6)/3.5).</td></tr>
<tr><td colspan="2">Start intravenous insulin infusion 1-2 hours after beginning intravenous fluid therapy, disconnect continuous subcutaneous insulin infusion (CSII).</td></tr>
<tr><td>Use a soluble insulin infusion at a dosage between 0.05 and 0.1 units/kg/hour. Do not give bolus doses of intravenous insulin.</td><td>0.05 units/kg/hour would be sufficient in most cases, but consider 0.1 units/kg/hr in severe DKA.</td></tr>
<tr><td colspan="2">When plasma glucose concentration falls below 14mmol/litre change fluids to 0.9% sodium chloride with 5% glucose and 40mmol/litre potassium chloride.

Once ketones <1.0mmol/l, consider switching from intravenous to subcutaneous insulin.</td></tr>
</table>

Figure 2 - Flowchart demonstrating the similarities and differences between the 2020 updated guidelines

88x137mm (150 x 150 DPI)

Guidelines	Severity of DKA	Dehydration
**NICE**	Mild-moderate – blood pH ≥7.1	5%
	Severe – blood pH <7.1	10%
**BSPED**	Mild - (venous pH 7.2-7.29 or bicarbonate <15mmol/l)	5%
	Moderate - (venous pH 7.1-7.19 or bicarbonate <10mmol/l)	7%
	Severe - (venous pH <7.1 or bicarbonate <5mmol/l)	10%

Table 1 - comparison of NICE and BSPED DKA classification and dehydration status

98x40mm (150 x 150 DPI)

BMJ Paediatrics Open

Update and harmonisation of guidance for the management of diabetic ketoacidosis in children and young people in the UK

Journal:	*BMJ Paediatrics Open*
Manuscript ID	bmjpo-2021-001079.R2
Article Type:	Review
Date Submitted by the Author:	14-May-2021
Complete List of Authors:	Rugg-Gunn, Charlotte; University of Liverpool School of Medicine; University of Liverpool Department of Women's and Children's Health<br>Deakin, Mark; Alder Hey Children's Hospital<br>Hawcutt, Daniel B; University of Liverpool, Department of Women's and Children's Health; NIHR Alder Hey Clinical Research Facility
Keywords:	Resuscitation, Diabetes

SCHOLARONE™
Manuscripts

I, the Submitting Author has the right to grant and does grant on behalf of all authors of the Work (as defined in the below author licence), an exclusive licence and/or a non-exclusive licence for contributions from authors who are: i) UK Crown employees; ii) where BMJ has agreed a CC-BY licence shall apply, and/or iii) in accordance with the terms applicable for US Federal Government officers or employees acting as part of their official duties; on a worldwide, perpetual, irrevocable, royalty-free basis to BMJ Publishing Group Ltd ("BMJ") its licensees and where the relevant Journal is co-owned by BMJ to the co-owners of the Journal, to publish the Work in this journal and any other BMJ products and to exploit all rights, as set out in our licence.

The Submitting Author accepts and understands that any supply made under these terms is made by BMJ to the Submitting Author unless you are acting as an employee on behalf of your employer or a postgraduate student of an affiliated institution which is paying any applicable article publishing charge ("APC") for Open Access articles. Where the Submitting Author wishes to make the Work available on an Open Access basis (and intends to pay the relevant APC), the terms of reuse of such Open Access shall be governed by a Creative Commons licence – details of these licences and which Creative Commons licence will apply to this Work are set out in our licence referred to above.

Other than as permitted in any relevant BMJ Author's Self Archiving Policies, I confirm this Work has not been accepted for publication elsewhere, is not being considered for publication elsewhere and does not duplicate material already published. I confirm all authors consent to publication of this Work and authorise the granting of this licence.

Confidential: For Review Only

Update and harmonisation of guidance for the management of diabetic ketoacidosis in children and young people in the UK

Charlotte EM Rugg-Gunn[1,2], Mark Deakin[3], Daniel B Hawcutt[2,4]

1: University of Liverpool Medical School, Liverpool, UK

2: Department of Women's and Children's Health, University of Liverpool, Liverpool, UK

3: Alder Hey Children's Hospital, Eaton Road, Liverpool, UK

4: NIHR Alder Hey Clinical Research Facility, Eaton Road, UK

Word Count: 2748

Figures: 2

Tables: 1

Reference count: 13

Keywords: Paediatrics, Diabetic Ketoacidosis, Resuscitation, Guideline, Diabetes

Correspondence to: Dr. Daniel B. Hawcutt, Department of Women's and Children's Health, University of Liverpool, Alder Hey Children's Hospital, Eaton Road, Liverpool, L12 2AP, UK; dhawcutt@liverpool.ac.uk

Confidential: For Review Only

ABSTRACT

Diabetic ketoacidosis (DKA) is a life-threatening complication of Type 1 Diabetes Mellitus. Careful and timely intervention is required to optimise glycaemic control and reduce the risk of mortality and devastating complications. Of these, cerebral oedema is the leading cause of death, with a mortality rate of approximately 25%. This article highlights the recent updates to UK fluid therapy guidelines for DKA and provides clinical context for the benefit of paediatricians and junior doctors in light of this new guidance.

KEY MESSAGES

- Updates to two UK guidelines in 2020 has improved concordance, with 0.9% sodium chloride and the Holliday-Segar formula used as gold-standard to calculate fluid maintenance requirement.
- Minor differences remain, such as the maximum weight for maintenance and the stratification of DKA or dehydration severity.
- Careful monitoring and adherence to these national guidelines is recommended and will hopefully contribute to a reduction in deaths secondary to cerebral oedema.
- Further research is required to evaluate the efficacy of these new guidelines, and better understand the pathophysiology and risk factors for developing cerebral oedema.

Confidential: For Review Only

INTRODUCTION

Diabetic ketoacidosis (DKA) is a potentially life-threatening metabolic complication of Type 1 Diabetes Mellitus (T1DM). Despite interventions to reduce the incidence of DKA, the National Paediatric Diabetes Audit (NPDA) for 2018/19 showed that overall 20.9% of all newly diagnosed patients in the United Kingdom (UK) presented in DKA, although there is considerable regional variability.(1)

DKA is characterised by uncontrolled hyperglycaemia, ketosis and subsequent metabolic acidosis. As highlighted in Figure 1, the clinical picture can develop insidiously due to a variable constellation of non-specific systemic signs and symptoms including polyuria, polydipsia, weight loss, fatigue, vomiting, and abdominal pain, ultimately leading to confusion, coma, and death if untreated (2, 3). Due to the ambiguity of the presenting clinical features in the early stages, delays in diagnosis are common.(4)

Globally, the International Society for Pediatric and Adolescent Diabetes (ISPAD) guidelines are widely used for management of paediatric DKA.(5) However, in the UK it is guided by the integrated care pathways of either the British Society for Paediatric Endocrinology and Diabetes (BSPED) or the National Institute for Health and Care Excellence (NICE). The BSPED guideline evolved from the NICE guideline in 2009 due to concerns over limited evidence that was used for its development. Whilst there were a number of dual-serving board members, the input and additional evidence by the BSPED special interest group in DKA was thought to increase its safety.(6) Both guidelines cover the diagnosis and management of Type 1 and Type 2 diabetes in children and young people (CYP) aged under 18.(7) Areas of particular benefit within a secondary care setting are centred on the requirements for fluid therapy, given the importance of resuscitation in preventing complications. Due to the difference in perceived risk of cerebral oedema (CO) due to rapid fluid administration, there was discordance between the NICE and BSPED 2015 guidelines.

Specifically, the NICE 2015 guidelines were more conservative with fluid administration, in order to avoid rapid changes in osmolality. The BSPED guidelines permitted a more generous fluid allowance, and this raised concerns about precipitating CO. The conflicting advice generated challenges in the emergency setting, with arbitrary site-specific preference rather than evidence-based medicine taking precedence. In addition to generating uncertainty of treatment pathways increasing the risk of clinical error, the regional variability hampered service evaluation and audit of clinical practice.

Unfortunately, there is little data to establish differences in their efficacy, especially surrounding the risk of CO in the paediatric population. However, inadequate resuscitation is likely to increase the risk of brain injury and thus must be avoided. Further research on a national scale is required to assess this.

This article aims to highlight the considerable alterations made to the two main UK guidelines. Alongside these updates, this article will provide clinical context to paediatricians and junior doctors who are likely to treat these vulnerable groups.

UPDATED GUIDANCE

In January 2020, the BSPED published new guidance for the management of children with DKA.(6) This integrated care pathway migrated further from the model of restrictive fluid replacement towards an even more flexible approach for resuscitative and maintenance fluids. The rationale for this was based on new evidence from the PECARN DKA Fluid trial, which suggested that there was no significant difference in outcomes between rapid and slower fluid administration.(8) Additionally, it showed that the initial conscious level was closely related to pH and weakly to age, but not to blood glucose or plasma sodium level.(8, 9) Whilst this appears to demonstrate that cerebral function is related to the severity of acidosis even in the absence of drivers of cerebral oedema, it does not negate previous evidence that fluid shift may also contribute towards cerebral oedema.(10, 11)

Confidential: For Review Only

Furthermore, there are clear risks associated with inadequate fluid replacement. A hypovolaemic state will result in systemic hypotension and ultimately, cerebral hypoperfusion which, particularly in the context of acidosis, will increase the risk of a brain injury.(6)

In 2008, following three deaths from cerebral oedema, the South Thames Retrieval Service introduced a "restrictive fluid" DKA guideline in the region. Subsequently, no deaths have been reported but this is insufficient evidence on which to make definitive recommendations or guidelines.(12)

More recently, in December 2020, NICE released updated guidance on the management of DKA in CYP which was broadly concordant with BSPED, particularly with respect to more liberal fluid restriction. This was prompted by new evidence identified by NICE's surveillance team in a review of 12 studies set to determine optimal fluid therapy in CYP with DKA. The fluid protocols followed in the PECARN study were fundamental to these updates,(8) as "neither the rate of administration nor the sodium chloride content of intravenous fluids significantly influenced neurologic outcomes in children with diabetic ketoacidosis".(8) Therefore, restrictions to the fluid administration which were recommended in the 2015 NICE guidance "were not necessarily required".(13) Additionally, as with the BSPED and ISPAD guidelines, the updated NICE guidance was amended to include the Holliday-Segar formula, used to calculate fluid maintenance.(6, 7) 2020 updates to both the BSPED and NICE guidelines have a more permissive fluid rate than in the previous DKA fluid therapy guidelines.

Both BSPED and NICE recommend the following fluid maintenance requirements, using 0.9% sodium chloride without added glucose, and the Holliday-Segar formula:

- 100ml/kg for the first 10kg (0-10kg body weight)
- 50ml/kg for the second 10kg (10-20kg body weight)
- 20ml/kg for each subsequent kg (>20kg body weight)
- When calculating the total fluid replacement, subtract any initial bolus volumes from the total fluid deficit (unless the child or young person is in shock)

For insulin and electrolyte replacement requirements, both recommend:(6, 7)

- The addition of 40mmol/l potassium chloride to all fluids (apart from the initial IV bolus) unless the patient is anuric or hyperkalaemic. Include this before starting the insulin infusion if hypokalaemia is observed at presentation.
- 0.9% sodium chloride should be used without added glucose unless the plasma glucose is <14mmol/l.
- Initially assess for presence of hyponatraemia, continue to monitor throughout treatment and treat as soon as blood glucose falls. It is important to monitor for both hyponatraemia and rapid hypernatraemia as these can both be signs of CO. Monitor sodium levels throughout treatment and calculate sodium initially to identify if hyponatraemia is present. This should be treated as soon as blood glucose falls. It is important to monitor as hyponatraemia and rapidly increasing sodium levels can both be signs of CO.
- Do not give IV sodium bicarbonate to CYP with DKA unless they have compromised cardiac contractility caused by life-threatening hyperkalaemia or severe acidosis, and you have discussed with paediatric intensivist.
- A soluble insulin infusion of between 0.05 and 0.1 units/kg/hour, 1-2 hours after IV fluids have been commenced. BSPED stated that the 0.05 units/kg/hr should be sufficient unless the patient is suffering from severe DKA or is an adolescent. If they are attached to a 'continuous subcutaneous insulin infusion', disconnect when starting IV insulin therapy. For patients already on long-acting insulin, it may be continued, and in new patients consider commencing long-acting subcutaneous insulin alongside IV. Similarly, the NICE guidelines state that continuing subcutaneous basal insulin in a child or young person who was using a basal insulin before DKA may be continued in discussion with a diabetes specialist.

Although these guidelines now have greater cohesion, a number of minor differences remain:(1, 6)

Confidential: For Review Only

- Regarding fluid replacement, NICE have included 2 levels of severity of DKA, whilst BSPED have subdivided the stratification to include 3 discrete categories for severity and subsequent dehydration status, as outlined in Table 1.
    - A difference remains between the maximum weight considered by the guidelines when calculating fluid deficit. Given that the maximum weight considered for each is 75kg for NICE and 80kg for BSPED, the maximum difference in deficit fluids could be 1,850ml (5% x 75kg = 3750ml for NICE compared to 7% x 80kg = 5600ml for BSPED).
- For patients who are clinically dehydrated, but not in shock, an IV 10 ml/kg bolus of 0.9% sodium chloride is recommended on admission. This should be administered over 30 minutes (NICE) or 60 minutes (BSPED). This volume is included in the fluid replacement when calculating the total fluid deficit. NICE recommend discussing with a paediatrician experienced in DKA management before giving more than one IV bolus. A second bolus should only be considered if needed to improve tissue perfusion and this must be done after reassessing their clinical status.
    - A delicate balance between increasing the risk of osmolar shift secondary to rapid fluid administration, with the risk of dehydration and subsequent CO due to slower fluid administration is the foundation for the initial discrepancy between the two guidelines. Guidance from the 2018 ISPAD consensus states that this should be administered between 30-60 minutes which falls between the two UK guidelines.(5) Therefore, aiming to administer the fluid rapidly but observing for signs of potential deterioration and adjusting the rate accordingly will provide the best outcome for patients.
- In the presence of hypovolaemic shock both NICE and BSPED recommend an IV 0.9% sodium chloride bolus of 20ml/kg. BSPED recommend repeating these infusions over 15 minutes to a maximum of 40ml/kg, before considering the introduction of inotropes, whilst NICE recommends administering it as soon as possible. In both guidelines, the bolus for shock is not subtracted from total fluid deficit.
    - Whilst the nomenclature varies between these two guidelines, in practice it is likely that these will represent similar time frames. Administering a 20ml/kg 0.9% sodium chloride bolus to treat shock as quickly as possible should provide the best therapy for the patient.
- When calculating fluid replacement, the NICE guidelines recommend a maximum weight of 75kg whereas BSPED recommend a maximum weight of 80kg or 97th centile weight for age (whichever is lower).
    - This discrepancy is unlikely to be encountered on a frequent basis in general paediatric practice. However, if the weight greatly exceeds 75kg it may be more appropriate for young people to be managed according to adult guidelines due to their increased body habitus. Additionally, identifying a CYP on the 97th centile can be difficult, especially in an emergency setting, and therefore this may be missed.

Figure 2 highlights the key similarities and differences between the updated 2020 guidelines.

**Guidelines**	**Severity of DKA**	**Dehydration**
NICE	Mild-moderate – blood pH ≥7.1<br>Severe – blood pH <7.1	5%<br>10%
BSPED	Mild - (venous pH 7.2-7.29 or bicarbonate <15mmol/l)<br>Moderate - (venous pH 7.1-7.19 or bicarbonate <10mmol/l)<br>Severe - (venous pH <7.1 or bicarbonate <5mmol/l)	5%<br>7%<br>10%

Table 1: comparison of NICE and BSPED DKA classification and dehydration status

Confidential: For Review Only

COMPARISON TO INTERNATIONAL GUIDANCE:

The new guidelines released by both NICE(7) and BSPED(6) read similarly to those of non-UK based origin. The 2018 consensus guidelines released by ISPAD(5) in conjunction with the European Society for Paediatric Endocrinology and the Lawson Wilkins Pediatric Endocrine Society (ESPE/LWPES) are used globally as standard guidelines for paediatric DKA management. Although the updated NICE and BSPED guidelines are very similar, as reviewed in Figure 2, the BSPED guidelines appear to be most similar to the ISPAD guidelines on fluid resuscitation and electrolyte balance, with very few differences between the two.

The most pertinent similarities between the BSPED and ISPAD guidelines include giving a bolus for shock (20mL/kg), using the Holliday-Segar formula to determine the maintenance fluid requirements, using a maximum of 80kg in weight for these calculations and stratifying the severity of DKA (into mild, moderate and severe) compared to the two classifications used by NICE (mild/moderate and severe).

In contrast to the NICE and BSPED guidelines which state a 0.9% sodium chloride solution must be used when considering fluid requirements, in the ISPAD guidance, either a 0.45% or 0.9% sodium chloride or a balanced salt solution (Ringer's lactate, Hartmann's solution or PlasmaLyte) may be used. This is secondary to recent findings from randomised control trials (RCTs) showing no difference in cerebral injury in patients rehydrated at different rates with either 0.45% or 0.9% sodium chloride. Additionally, for those not in shock, these fluids should be infused over 30 to 60 minutes which falls between the two 2020 guidelines (NICE states to give fluids over 30 minutes, BSPED states over 60 minutes). Regarding the fluid bolus for those in shock, as with the NICE 2020 guidelines, the ISPAD guidelines state that it should be given "as soon as possible". Conversely, BSPED says this should be over 15 minutes. However, in practice this difference in nomenclature is likely to be negligible. Also in agreement with the NICE 2020 guidelines, the ISPAD guidelines recommend the prescription of insulin at between 0.05 and 0.1 units/kg/hour, where 0.05 units/kg/hour is usually sufficient in mild DKA. When assessing glucose introduction, the ISPAD guidance recommends that healthcare professionals "consider adding glucose before 17 mmol if falling rapidly. Otherwise 5% glucose should be added when the plasma glucose falls to approximately 14 to 17 mmol/L/". This is in contrast to the NICE and BSPED guidelines which only introduce 5% glucose when it has fallen below 14mmol/L.

The Royal Children's Hospital in Melbourne (RCH) Clinical Practice Guidelines for Diabetic Ketoacidosis, last updated in November 2018, have been adapted for statewide use with the support of the Victorian Paediatric Clinical Network. Although broadly similar, there are a few distinct differences between the RCH guidelines and those of NICE, BSPED and ISPAD. For those in shock, a 10mL/kg 0.9% sodium chloride bolus can be given, half the volume of 20mL/kg recommended by the other guidelines. Although initial fluid rates are based on a degree of dehydration (with the RCH stratifications varying from the other guidelines – mild = <4%, moderate = 4-7% and severe = >7%), maintenance fluids are not calculated using the Holliday-Segar formula. Instead a detailed table is used, with the fluid requirements classified according to the different degrees of dehydration up to a maximum of 70kg.

However, as with the other guidelines, 40mmol/L of potassium chloride is added to the fluid, as long as the child is not anuric or a serum potassium ≥5.5 mmol/L. If required, these can be increased to a maximum of 60mmol/L, which is not specified in the other guidelines. Additionally, 5% glucose is added if the blood glucose is ≤15 mmol/L, which is comparable, although this can be increased to 10% glucose if blood glucose is falling rapidly or ≤5 mmol/L. Insulin infusion rate of 0.1 units/kg/hour is recommended unless treating children under 5 years, children undergoing inter-hospital transfer (where limited access to biochemical monitoring) or children with blood glucose <15 mmol/L at the time of commencement of the insulin infusion. This is placed at the upper end of the bracket of the other guidelines, where 0.1 units/kg/hour is recommended for those with severe DKA, adolescence or unresolved ketosis.

CONCLUSIONS AND RECOMMENDATIONS:

The convergence of the NICE and BSPED guidelines is a welcome development in the emergency management of paediatric DKA. This will provide much needed clarity to the treating clinicians, reducing regional variation and decreasing the potential for error. Despite the pathophysiology of CO remaining uncertain, fluid therapy must be carefully controlled, as inadequate resuscitation is likely to increase the risk of brain injury and thus must be avoided.

There is emerging evidence for a more judicious approach in the management of DKA(9) and the NICE and BSPED guidelines are now largely concordant in this regard. A number of minor differences in the guidelines remain but these are unlikely to profoundly influence patient management or outcomes. As both guidelines have changed considerably since their conception, and now have only minor differences, it could be argued that there is no benefit of two guidelines. The existence of two guidelines in the UK is primarily historical, with both guideline-producing groups having good reason to be interested in this area. However, the UK would, in our opinion, be better served with a single set of guidance to avoid confusion and harmonise practice.

Despite this, it is imperative that the incidence of CO continues to be monitored following a change to the guidelines and, in addition, this may offer insights into the underlying pathophysiological basis and effective preventative measures.

In the meantime, for junior clinicians treating DKA in CYP, close supervision by experienced paediatricians, careful monitoring and adherence to a single-set of evidence-based guidelines are likely to translate into better outcomes for this challenging condition.

Author contributorship: Concept MD, writing CRG, DH, MD, editing DH, MD

Competing interests and funding: none declared

I, the Submitting Author has the right to grant and does grant on behalf of all authors of the Work (as defined in the below author licence), an exclusive licence and/or a non-exclusive licence for contributions from authors who are: i) UK Crown employees; ii) where BMJ has agreed a CC-BY licence shall apply, and/or iii) in accordance with the terms applicable for US Federal Government officers or employees acting as part of their official duties; on a worldwide, perpetual, irrevocable, royalty-free basis to BMJ Publishing Group Ltd ("BMJ") its licensees and where the relevant Journal is co-owned by BMJ to the co-owners of the Journal, to publish the Work in BMJ Paediatrics Open and any other BMJ products and to exploit all rights, as set out in our licence.

The Submitting Author accepts and understands that any supply made under these terms is made by BMJ to the Submitting Author unless you are acting as an employee on behalf of your employer or a postgraduate student of an affiliated institution which is paying any applicable article publishing charge ("APC") for Open Access articles. Where the Submitting Author wishes to make the Work available on an Open Access basis (and intends to pay the relevant APC), the terms of reuse of such Open Access shall be governed by a Creative Commons licence – details of these licences and which Creative Commons licence will apply to this Work are set out in our licence referred to above.

Confidential: For Review Only

References:

1. Schnabel A, Hedrich CM. Childhood Vasculitis. Frontiers in pediatrics. 2018;6:421.
2. Wolfsdorf J, Glaser N, Sperling MA. Diabetic Ketoacidosis in Infants, Children, and Adolescents: A consensus statement from the American Diabetes Association. Diabetes Care. 2006;29(5):1150-9.
3. Ribero S, Sciascia S, Borradori L, Lipsker D. The Cutaneous Spectrum of Lupus Erythematosus. Clinical reviews in allergy & immunology. 2017;53(3):291-305.
4. Unnikrishnan R, Shah VN, Mohan V. Challenges in diagnosis and management of diabetes in the young. Clinical Diabetes and Endocrinology. 2016;2(1).
5. Wolfsdorf JI, Glaser N, Agus M, Fritsch M, Hanas R, Rewers A, et al. ISPAD Clinical Practice Consensus Guidelines 2018: Diabetic ketoacidosis and the hyperglycemic hyperosmolar state. Pediatr Diabetes. 2018;19 Suppl 27:155-77.
6. Integrated care pathway for the management of children and young people with Diabetic Ketoacidosis British Society for Paediatric Endocrinology and Diabetes; 2020 [Available from: https://www.bsped.org.uk/media/1742/dka-icp-2020-v1_1.pdf.
7. Diabetes (type 1 and type 2) in children and young people: diagnosis and management: NICE guideline NG18; 2020 [Available from: https://pathways.nice.org.uk/pathways/diabetes-in-children-and-young-people/diabetic-ketoacidosis-in-children-and-young-people#content=view-node%3Anodes-recognition-referral-and-diagnosis.
8. Kuppermann N, Ghetti S, Schunk JE, Stoner MJ, Rewers A, McManemy JK, et al. Clinical Trial of Fluid Infusion Rates for Pediatric Diabetic Ketoacidosis. N Engl J Med. 2018;378(24):2275-87.
9. Edge JA, Roy Y, Bergomi A, Murphy NP, Ford-Adams ME, Ong KK, et al. Conscious level in children with diabetic ketoacidosis is related to severity of acidosis and not to blood glucose concentration. Pediatr Diabetes. 2006;7(1):11-5.
10. Duck SC, Wyatt DT. Factors associated with brain herniation in the treatment of diabetic ketoacidosis. J Pediatr. 1988;113(1 Pt 1):10-4.
11. Arieff AI, Kleeman CR. Studies on mechanisms of cerebral edema in diabetic comas. Effects of hyperglycemia and rapid lowering of plasma glucose in normal rabbits. J Clin Invest. 1973;52(3):571-83.
12. Lillie J, Boot E, Tibby SM, McDougall M, Nyman A, Griffiths B, et al. Management of fluids in paediatric diabetes ketoacidosis: concerns over new guidance. Arch Dis Child. October 2020;105:1019-20.
13. Diabetes (type 1 and type 2) in children and young people: diagnosis and management. Evidence reviews for fluid therapy for the management of diabetic ketoacidosis. Guideline version (Draft). National Institute for Health and Care Excellence; 2020.

Confidential: For Review Only

Clinical history:

- Polyuria/polydipsia
- Weight loss
- Abdominal pain
- Weakness
- Vomiting
- Confusion

Clinical signs:

- Dehydration
- Kussmaul breathing
- Ketotic smell
- Lethargy, drowsiness

Biochemistry:

- Hyperglycaemia (>11mmol/L)
- Acidaemia (pH<7.3)
- Ketosis (blood ketones >3mmol/L or urine ketones ++)

Potential triggers:

- Infection e.g. viral
- Interruption to insulin therapy e.g. pump malfunction or omitted doses
- Physical or emotional trauma
- Pregnancy
- Drug interactions including alcohol and particularly cocaine

Figure 1: Key signs and symptoms of Diabetic Ketoacidosis, adapted from (1)

32x68mm (300 x 300 DPI)

<table>
<tr><th>NICE</th><th>BSPED</th></tr>
<tr><td colspan="2">If in shock – 20ml/kg bolus of 0.9% sodium chloride.

Not in shock – 10ml/kg bolus of 0.9% sodium chloride.

When calculating the total fluid replacement, subtract any initial bolus volumes from the total fluid deficit (if in shock – do not include the bolus volumes).</td></tr>
<tr><td>In shock – bolus given as soon as possible.

Not in shock – bolus given over 30 minutes.</td><td>In shock – bolus given over 15 minutes.

Reassess and further boluses of 10ml/kg up to a total of 40ml/kg – then consider inotropes.

Not in shock – bolus given over 60 minutes.</td></tr>
<tr><td colspan="2">Fluid requirement = Deficit + Maintenance

Hourly rate = ((Deficit – any initial bolus)/48 hours) + Maintenance per hour</td></tr>
<tr><td>Mild to moderate DKA (blood pH 7.1 or above) = 5% dehydration.

Severe DKA (blood pH below 7.1) = 10% dehydration.</td><td>Mild DKA (venous pH 7.2-7.29 or bicarbonate <15 mmol/l) = 5% dehydration.

Moderate DKA (venous pH 7.1-7.19 or bicarbonate <10mmol/l) = 7% dehydration.

Severe DKA (venous pH less than 7.1 or bicarbonate <5 mmol/l) = 10% dehydration.</td></tr>
<tr><td colspan="2">Fluid maintenance: 0.9% sodium chloride without added glucose, Holliday-Segar formula.

(100ml/kg for the first 10kg, 50ml/kg for the second 10kg, and 20ml/kg for each subsequent kg)

Given over 48 hours</td></tr>
<tr><td>Maximum weight of 75kg in the Holliday-Segar formula</td><td>Maximum weight of 80kg or 97th centile weight for age (whichever is lower) in the Holliday-Segar formula</td></tr>
<tr><td colspan="2">All fluids (except any initial boluses) contain 40mmol/litre (or 20mmol/500ml) potassium chloride, unless they have renal failure or hyperkalaemia. Do not delay potassium replacement.</td></tr>
<tr><td colspan="2">Monitor serum sodium levels – they should rise as DKA is treated. Falling serum sodium is a sign of possible cerebral oedema. Use corrected sodium values = measured sodium + ((glucose – 5.6)/3.5)</td></tr>
<tr><td colspan="2">Start intravenous insulin infusion 1-2 hours after beginning intravenous fluid therapy, disconnect continuous subcutaneous insulin infusion (CSII).</td></tr>
<tr><td>Use a soluble insulin infusion at a dosage between 0.05 and 0.1 units/kg/hour. Do not give bolus doses of intravenous insulin.</td><td>0.05 units/kg/hour would be sufficient in most cases, but consider 0.1 units/kg/hr in severe DKA.</td></tr>
<tr><td colspan="2">When plasma glucose concentration falls below 14mmol/litre, change fluids to 0.9% sodium chloride with 5% glucose and 40mmol/litre potassium chloride.

Once ketones <1.0mmol/l, consider switching from intravenous to subcutaneous insulin.</td></tr>
</table>

Figure 2 - Flowchart demonstrating the similarities and differences between the 2020 updated guidelines

84x106mm (300 x 300 DPI)
